# Poisons on Different Animals

**Published:** 1871-12

**Authors:** 


Varying Effects of Poisons on Different Animals.—It is a well-known fact that what is poisonous to one animal may be taken by another with entire impunity. In illustration of this proposition, we are informed that strychnine, so fatal to most animals, may be eaten by certain species of monkeys with perfect safety. In the case of an East India monkey, known as the Lungoor (*Presbytis entellus*), one grain was first concealed in a piece of cucumber, which was eaten by the animal with no apparent effect. Three grains were afterward given, and with the same result. To test the strychnine used, three grains were administered to a dog, which proved almost immediately fatal. Another Indian monkey, known as the pouch cheek monkey, has been found to be more susceptible than the Lungoor, but not so much so as the dog.

It is also stated that pigeons can take opium in large quantities with no injurious consequence; goats, tobacco; and rabbits, belladonna, strammonium and hyoscyamus.—*Agr. Reporter.*

